# Dislocation of the Lower End of the Ulna Forwards

**Published:** 1872-12

**Authors:** John H. Gilman

**Affiliations:** Harv.


### DISLOCATION OF THE LOWER END OF THE ULNA FORWARDS.

BY JOHN H. GILMAN, M.D., Harv.

This rare accident occurred to M. B. D., July 23, 1872, while engaged in fixing a loom in the boot mill of this city. The left forearm, being between the spokes of a wheel when the loom was set in motion, was violently turned outwards. On examination, the hand was found in a state of fixed supination; on looking at the back of the wrist, there was a marked depression of the ulnar portion, and an unusual prominence of the corresponding portion on the frontal aspect of the wrist. Reduction was effected by grasping the hand and making extension (counter extension being made at the elbow), slightly pronating the hand and raising the bone into place with the right hand. *Boston Medical and Surgical Journal.*

Lowell, August 6, 1872.

